# Resourcization of Argillaceous Limestone with Mn_3_O_4_ Modification for Efficient Adsorption of Lead, Copper, and Nickel

**DOI:** 10.3390/toxics12010072

**Published:** 2024-01-15

**Authors:** Deyun Li, Yongtao Li, Shuran He, Tian Hu, Hanhao Li, Jinjin Wang, Zhen Zhang, Yulong Zhang

**Affiliations:** 1 School of Environmental Science and Engineering, Shaanxi University of Science & Technology, Xi’an 710021, China; deyun_94@163.com (D.L.); yongtao@scau.edu.cn (Y.L.); lihanhao_happy@126.com (H.L.); 2 College of Natural Resources and Environment, Joint Institute for Environmental Research & Education, South China Agricultural University, Guangzhou 510642, China; hut0429@scau.edu.cn (T.H.); wangjinjin@scau.edu.cn (J.W.); zzhangal@scau.edu.cn (Z.Z.); 3 College of Resource and Environment, Yunnan Agricultural University, Kunming 650201, China; shuran@ynau.edu.cn

**Keywords:** manganese oxides, argillaceous limestone, adsorption mechanism, heavy metal, competitive adsorption

## Abstract

Argillaceous limestone (AL) is comprised of carbonate minerals and clay minerals and is widely distributed throughout the Earth’s crust. However, owing to its low surface area and poorly active sites, AL has been largely neglected. Herein, manganic manganous oxide (Mn_3_O_4_) was used to modify AL by an in-situ deposition strategy through manganese chloride and alkali stepwise treatment to improve the surface area of AL and enable its utilization as an efficient adsorbent for heavy metals removal. The surface area and cation exchange capacity (CEC) were enhanced from 3.49 to 24.5 m^2^/g and 5.87 to 31.5 cmoL(+)/kg with modification, respectively. The maximum adsorption capacities of lead (Pb^2+^), copper (Cu^2+^), and nickel (Ni^2+^) ions on Mn_3_O_4_-modified argillaceous limestone (Mn_3_O_4_–AL) in mono-metal systems were 148.73, 41.30, and 60.87 mg/g, respectively. In addition, the adsorption selectivity in multi-metal systems was Pb^2+^ > Cu^2+^ > Ni^2+^ in order. The adsorption process conforms to the pseudo-second-order model. In the multi-metal system, the adsorption reaches equilibrium at about 360 min. The adsorption mechanisms may involve ion exchange, precipitation, electrostatic interaction, and complexation by hydroxyl groups. These results demonstrate that Mn_3_O_4_ modification realized argillaceous limestone resourcization as an ideal adsorbent. Mn_3_O_4_-modified argillaceous limestone was promising for heavy metal-polluted water and soil treatment.

## 1. Introduction

Over the years, large amounts of wastewater containing lead (Pb^2+^), copper (Cu^2+^), nickel (Ni^2+^), and other heavy metal ions have been discharged into surface and underground water. These heavy metals, originating from metal electroplating, mining, battery manufacturing, chemical industries, and other industries [1], are widely recognized as one of the most severe threats to human health, animal well-being, and plants, due to their carcinogenicity, toxicity, non-degradability, persistence in the environment, and ability to bioaccumulate within food chains [2,3,4]. Consequently, there is an urgent need for effective removal of heavy metal ions from wastewater.

One of the most efficient techniques for remediating water contaminated with heavy metals is adsorption due to its advantages, such as availability, cost-effectiveness, and ease of operation [5,6]. The key aspect of the adsorption technique is to select an eco-friendly, inexpensive, and efficient adsorbent. Natural clays, along with other minerals, are considered green adsorbents due to their affordability and abundance as a natural resource [7]. At present, various studies have focused on utilizing minerals for wastewater treatment. Du et al. (2016) investigated the absorption behavior of Pb^2+^ and Cd^2+^ in water using a bacteria–montmorillonite composite material [8]. Zhao et al. (2022) synthesized a silicate-based composite material and demonstrated its remarkable capacity to adsorb Cd^2+^ (63.80 mg/g) [9].

Argillaceous limestone (AL), comprised of carbonate and clay minerals, is widely distributed throughout the Earth’s crust. Due to the precipitation properties of the carbonate minerals and the high cation exchange capacity of the clay minerals, both are effective in removing heavy metals [7,10,11]. However, research on AL as a heavy metal adsorbent remains relatively limited [12]. This is primarily attributed to its low content of functional components, low surface area, and poor active sites [13]. Therefore, it is necessary to modify or functionalize AL before it can be applied as a heavy metal adsorbent. Additionally, AL commonly coexists with grey limestone as an associated mineral. After extracting grey limestone for building materials purposes, AL is often abandoned [14]. Therefore, AL has the advantage of low cost. The modification of AL as an adsorbent for heavy metal removal holds great significance for resource utilization.

Manganese oxide-modified adsorbents have attracted researchers’ attention due to their high electronegativity, surface area, and other unique physicochemical properties [6,15,16,17,18,19]. Moreover, manganese oxides can be firmly loaded onto silica-based surfaces [18]. Additionally, manganese oxides are abundant in geological reserves and therefore, also serve as a low-cost material [19]. To date, reports have confirmed the potential application of Mn_3_O_4_ as a good adsorbent to remove metal ions from water. Lingamdinne et al. (2022) found that Mn_3_O_4_-anchored reduced graphene oxide had significant adsorption efficiency for lead and chromium ions and the maximum sequestration capacities were 130.28 and 138.51 mg/g, respectively [6]. In addition, the research of Zou et al. (2016) showed that the maximum adsorption of antimony ions on Mn_3_O_4_-modified reduced graphene oxide was 151.84 mg/g [17]. Lee et al. prepared Mn_3_O_4_-coated activated carbon (Mn_3_O_4_/AC) for use in the removal of Pb and Cu ions, and showed that the adsorption capacities of Mn_3_O_4_/AC were enhanced 2.2 and 6.1 times for Pb and Cu ions, respectively, compared with pure active carbon [15]. However, to the best of our knowledge, limited studies have been conducted on the adsorption of heavy metals by the Mn_3_O_4_-modified AL (Mn_3_O_4_–AL). Previous research has indicated that environmental factors, such as pH, ionic strength, and organic matter, can influence the adsorption capacity of materials. It has been demonstrated that an increase in pH significantly enhances the adsorption of heavy metals [6,20]. Nonetheless, the effect of coexisting ions on adsorption is inconsistent and may hinder or promote the adsorption process [21]. In addition, it has been reported that humic acid can affect the adsorption behavior of heavy metal ions by mineral or synthetic adsorbents [22]. Therefore, it is crucial to investigate the effects of these environmental factors on adsorption for practical applications.

In this study, we modified AL with Mn_3_O_4_ using an in-situ deposition strategy through a manganese chloride (MnCl_2_) and alkali stepwise treatment to improve its adsorption capacity. Consequently, the purposes of this study were to: (1) synthesize and characterize the Mn_3_O_4_–AL; (2) explore the capacity of Mn_3_O_4_–AL to adsorb Pb^2+^, Cu^2+^, and Ni^2+^ in mono- or multi-heavy metal systems; (3) examine the impact of environmental factors (pH, ionic strength, and humic acid) on adsorption; and (4) clarify the adsorption mechanism of the adsorption process. The results obtained from this research are expected to provide valuable insights into understanding the ability of Mn_3_O_4_–AL to adsorb Pb^2+^, Cu^2+^, and Ni^2+^ and contribute towards resource utilization of AL. This work indicated that the Mn_3_O_4_–AL was a promising candidate for efficient heavy metal removal.

## 2. Materials and Methods

### 2.1. Materials and Chemicals

Argillaceous limestone (AL) was collected from the lower Cambrian Mantou formation in the Dabeiwang area of Jiangsu province, China. Our previous research showed that the raw minerals are mainly composed of carbonate minerals, accounting for 41.8%. In addition, clay minerals account for around 12.2% [23].

Sodium nitrite (NaNO_3_), calcium nitrite (Ca(NO_3_)_2_), nitric acid (HNO_3_, 70% (*w*/*w*)), sodium hydroxide (NaOH), hydrochloric acid (HCl, 37% (*w*/*w*)), manganese chloride (MnCl_2_), copper nitrate pentahydrate (Cu(NO_3_)_2_·5H_2_O), zinc nitrate hexahydrate (Zn(NO_3_)_2_·6H_2_O), and cadmium nitrate tetrahydrate (Cd(NO_3_)_2_·4H_2_O), lead nitrate (Pb(NO_3_)_2_), and nickel nitrate hexahydrate (Ni(NO_3_)_2_·6H_2_O, 99.99%) of analytical grade were purchased from Aladdin Reagent Database Inc. (Shanghai, China). Humic acid (HA) was purchased from Sigma-Aldrich (St Louis, MO, USA).

### 2.2. Modification of Argillaceous Limestone

**Figure 1:**
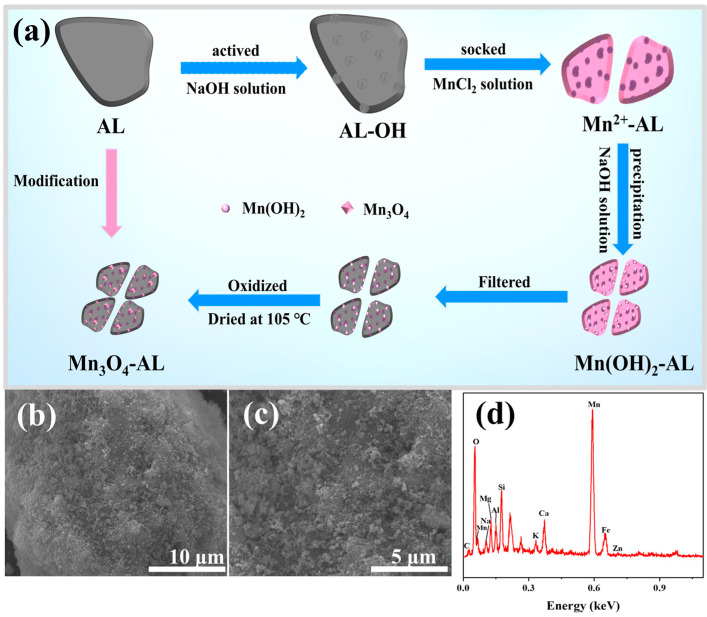
Schematic route of the modification of Mn_3_O_4_–AL (**a**). SEM (**b**,**c**) and EDS (**d**) images of Mn_3_O_4_–AL.

The modification process for argillaceous limestone (AL) was carried out following the previous study of Sarı et al. (2012) [19]. The synthesis pathway of modifying argillaceous limestone with Mn_3_O_4_ is shown in Figure 1a. Detailed synthesis steps are as follows: 20 g AL was activated with 80 mL of 6 mol/L NaOH at 90 °C for 2 h. Next, the obtained mixture was added to a 100 mL solution of 2.5 mol/L MnCl_2_ (with the pH adjusted to 2.0 using 1 M HCl), and placed at room temperature (25 °C) for 10 h. The mixture was filtered and the resulting precipitate was mixed with 40 mL of a 6 mol/L NaOH solution at 25 °C for 10 h to obtain a manganese hydroxide (Mn(OH)_2_) precipitate. The mixture was exposed to the air to oxidize the manganese hydroxide. Then, the modified argillaceous limestone was washed with deionized water and dried at 105 °C.

### 2.3. Batch Adsorption Experiments

Stock solutions of 1000 mg/L Pb^2+^, Cu^2+^, and Ni^2+^ were prepared using Pb(NO_3_)_2_, Cu(NO_3_)_2_·5H_2_O, Ni(NO_3_)_2_·6H_2_O. The desired pH of the suspensions was controlled using 0.1 mol/L NaOH or HNO_3_. NaNO_3_ and Ca(NO_3_)_2_ were used as background electrolytes to adjust the ionic strength of the suspensions. Adsorption kinetic experiments were conducted by adding 0.0075 g of the Mn_3_O_4_–AL into 50 mL solutions containing either 50 mg/L Pb^2+^, Cu^2+^, or Ni^2+^ or all three metals. The mixtures were shaken at 25 °C for 0–1080 min. To investigate the impact of the initial concentration and to compare the mono- and multi-heavy metal adsorption capacity of the Mn_3_O_4_–AL, adsorption isotherm experiments were carried out in multi-heavy metal and single-heavy metal solutions with initial concentrations in the range of 1–200 mg/L. Various concentrations of environmental factors, including pH (2.0, 3.0, 4.0, 5.0, 6.0), ionic strength (sodium ions(Na^+^), calcium ions (Ca^2+^), chloride ions (Cl^−^), and sulfate ions (SO_4_^2−^); 0, 0.01, and 0.1 mol/L), and humic acid (HA) (0, 0.1, 0.5, 1, 3, 5, 8, 10, 15, 20, 30 mg/L), were individually controlled to investigate their influence on heavy metal adsorption. In the isotherm and environmental factor experiments, the mixture was shaken for 12 h, and the other details were the same as in the adsorption kinetic experiments.

In addition, to explore the adsorption capacity of the Mn_3_O_4_–AL in the natural environment, lake water was used as the background water to simulate natural environment conditions. The lake water was collected from Zhaoyang Lake, at the South China Agricultural University, Guangdong Province, China. The pH of the lake water was 7.28, and the dissolved organic carbon (DOC) was 4.02 mg/L. The initial concentrations of total Cd, Pb, Cu, Ni, and Zn of the lake water were 0.08, 1.36, 1.88, 2.06, and 1.22 μg/L, respectively, which was below the environmental quality standards for surface water in China (GB 3838-2002) [24]. The mixed solution was prepared using Pb(NO_3_)_2_, Cu(NO_3_)_2_·5H_2_O, Ni(NO_3_)_2_·6H_2_O, Zn(NO_3_)_2_·6H_2_O, and Cd(NO_3_)_2_·4H_2_O. The initial concentrations of Pb^2+^, Cu^2+^, Ni^2+^, Cd^2+^, and Zn^2+^ in the multi-metal solution were each 50 mg/L. The mixture was shaken for 12 h, and other details were the same as in the adsorption kinetic experiments.

After shaking, all samples were immediately filtered with 0.22 µm filters. The concentrations of Pb^2+^, Cu^2+^, and Ni^2+^ were determined using flame atomic absorption spectroscopy (AAS) (Z2300, Hitachi, Tokyo, Japan). To ensure the reproducibility and accuracy of data, all of the experiments in this study were performed in triplicate.

The pseudo-first-order and pseudo-second-order models were applied to fit the adsorption kinetics of Pb^2+^, Cu^2+^, and Ni^2+^ onto the Mn_3_O_4_–AL (Equations (1) and (2)) [21].

(1) $$\log\left(Q_{e}{-Q}_{t}\right){=\log\;Q}_{e}-\frac{k_{1}t}{2.303}$$

(2) $$\frac{t}{Q_{t}}=\frac{1}{k_{2}Q_{e}^{2}}+\frac{t}{Q_{e}}$$

where *Q_e_* and *Q_t_* are the adsorption capacity at equilibrium and time *t* (mg/g), respectively. *k*_1_ is the rate constant of pseudo-first-order (min^−1^), and *k*_2_ is the rate constant of pseudo-second-order (g/(mg min)).

In addition, the Langmuir and Freundlich models (Equations (3) and (4)) were used to simulate the adsorption isotherm data [6].

(3) $$\frac{C_{e}}{Q_{e}}=\frac{1}{K_{L}Q_{m}}\times \frac{C_{e}}{Q_{m}}$$

(4) $${\log\;Q}_{e}{=\log\;K}_{F}+\frac{1}{n}{\log\;C}_{e}$$

where, *C_e_* is the equilibrium concentration of the heavy metal ions in solution (mg/L). *Q_e_* and *Q_m_* are the equilibrium and maximum adsorption capacity, respectively (mg/g). *K_L_* is the Langmuir constant (L/mg), *K_F_* is the Freundlich constant (mg^1−(1/n)^ L^1/n^/g), and 1/*n* is the heterogeneity factor.

### 2.4. Characterization Method

The mineralogical compositions of the Mn_3_O_4_–AL before and after adsorption were analyzed by X-ray diffraction (XRD) (D8 Advance, Bruker, Germany). The morphologies of the Mn_3_O_4_–AL before and after adsorption were characterized using scanning electron microscopy (SEM) and X-ray energy dispersive spectrometry (EDS) (Quanta FEG 650, FEI, Houston, TX, USA). The surface area of the Mn_3_O_4_–AL was measured using a gas sorption analyzer (Gemini VII 2390 V1.03, Micromeritics Instrument Corp, Norcross, GA, USA) by the Brunauer–Emmett–Teller (BET) method. A FT-IR instrument (Nicolet iS10, Thermo Fisher, Waltham, MA, USA) was used to obtain the change of functional groups before and after adsorption. Surface chemical compositions of the Mn_3_O_4_–AL before and after adsorption were evaluated by X-ray photoelectron spectroscopy (XPS) (ESCALAB 250Xi, Thermo Fisher, USA). The zeta potential of the Mn_3_O_4_–AL under different background solutions was analyzed by a dynamic light scattering analyzer (Nano-ZS90 Zetasizer, Malvern Instruments, Malvern, UK). The total organic carbon (TOC) of the Mn_3_O_4_–AL was determined with a TOC analyzer (Vario TOC, Elementar, Langenselbold, Germany). The cation exchange capacity (CEC) of the Mn_3_O_4_–AL was determined based on He et al. (2018) [23]. The pH and dissolved organic carbon (DOC) were determined using the procedure of Zhang et al. (2017) [21].

### 2.5. Statistical Analysis

Data are presented as the mean ± standard deviation (SD). All data were statistically assessed by one-way ANOVA with the minimum level of significance set at *p* < 0.05. In addition, all of the functional graphs were produced using OriginPro 2021 software.

## 3. Results and Discussion

### 3.1. Characterization of Mn_3_O_4_ Modified Argillaceous Limestone

To obtain a satisfactory and economical adsorbent, raw argillaceous limestone was modified by in-situ deposition of Mn_3_O_4_ from a MnCl_2_ precursor solution with alkalized treatment. The morphology and chemical composition of the Mn_3_O_4_–AL were analyzed using SEM and EDS (Figure 1b,c). Previous research indicated that raw argillaceous limestone exhibited dense massive structures [23]. However, after Mn_3_O_4_ modification, the surface of the argillaceous limestone became fluffy and featured the distribution of numerous small granular particles. This transformation can be attributed to the acidic atmosphere created by the strong acid and weak alkali salt MnCl_2_, which dissolved carbonates. Consequently, argillaceous limestone was broken down into smaller particles, leading to an increase in its specific surface area. This observation was further supported by a significant rise in the specific surface area from 3.49 to 24.5 m^2^/g after modification (Table S1). EDS spectra results revealed that the Mn_3_O_4_–AL mainly consisted of manganese (Mn), silicon (Si), oxygen (O), calcium (Ca), magnesium (Mg), aluminum (Al), potassium (K), and carbon (C) (Figure 1c). Notably different from our previous research on AL [23], there was a distinct peak corresponding to Mn present in the EDS of the Mn_3_O_4_–AL. In contrast, there is no Mn peak in AL. These findings indicated the successful loading of Mn onto raw argillaceous limestone.

**Figure 2:**
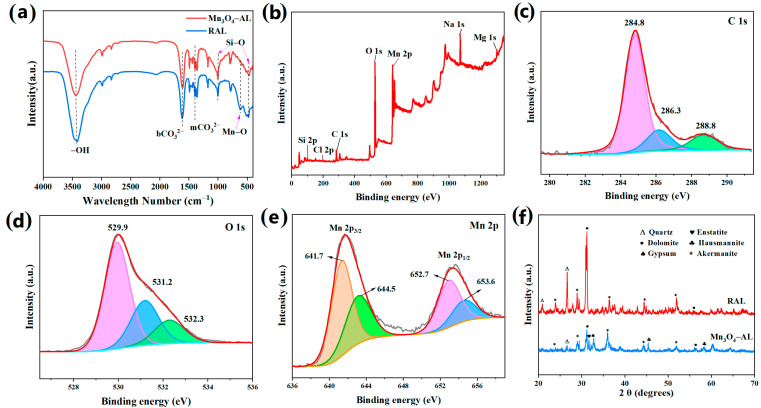
FT-IR spectra of Mn_3_O_4_–AL and AL (**a**). XPS spectra of wide scan, O 1s, C 1s, and Mn 2p of Mn_3_O_4_–AL after modification (**b**–**e**). XRD patterns spectra of Mn_3_O_4_–AL and AL (**f**).

The FT-IR spectra of AL and the Mn_3_O_4_–AL are depicted in Figure 2a. For the AL, the broad band at 3437 cm^−1^ can be attributed to the stretching vibration of –OH groups [25,26]. The peaks at 1396 cm^−1^ and 1615 cm^−1^ were the characteristic peaks for bCO_3_^2−^ and mCO_3_^2−^, respectively [21,27]. Negatively charged functional groups (–OH and COO–) existed on the surface of AL, which facilitates the adsorption of heavy metal ions through electrostatic adsorption and complexation reactions [21]. The bands at 1008 cm^−1^ and 521 cm^−1^ refer to Si–O symmetric stretching [28,29]. After modification, the signals of –OH and COO– were still maintained in the Mn_3_O_4_–AL curve. The intensity of CO_3_^2−^ peak at 1473 cm^−1^ became weaker, indicating partial dissolution of the carbonate mineral [30]. This is consistent with the result of the increasing specific surface area of the Mn_3_O_4_–AL. Additionally, a new peak appeared at 635 cm^−1^, which is consistent with the Mn–O–Mn stretching vibration [31,32,33]. It demonstrated that Mn was loaded onto the argillaceous limestone in the form of manganese oxide.

XPS was applied to analyze the chemical states of the Mn_3_O_4_–AL (Figure 2). The C 1s spectrum exhibited three characteristic peaks at 284.8, 286.3, and 288.8 eV (Figure 2c), corresponding to C–C, C–O–H/C–O–C, and O–C=O, respectively. Combined with the FT-IR result mentioned earlier, it can be concluded that the main form of C was carbonate. Three peaks were observed at 529.9, 531.2, and 532.3 eV in terms of O 1s high resolution (Figure 2d), corresponding to lattice oxygen (Mn–O–Mn), –OH, and C=O [34,35], respectively. The peaks at 641.7 eV and 652.7 eV in the detailed spectra of Mn 2p (Figure 2e) were attributed to 2p_3/2_ and 2p_1/2_ Mn (III), and the peaks at 644.5 eV and 653.6 eV were attributed to Mn(II) [17,36,37]. Combined with the results of FT-IR, we can speculate that Mn exists in the form of Mn_3_O_4_ in the Mn_3_O_4_–AL [35].

The mineralogical compositions of AL before and after modification with Mn_3_O_4_ were determined by XRD (Figure 2f). The XRD curve depicted that AL was mainly comprised of dolomite (CaMg(CO_3_)_2_) (2θ = 31.20°) (PDF No. 03-0864) and quartz (PDF No. 82-0513). The peaks at 2θ = 28.98° and 35.98° were regarded as akermanite (Ca_2_MgSi_2_O_7_) (PDF No. 35-0592). With the treatment of NaOH and MnCl_2_ sequentially, the location of the dominating diffraction peaks in the Mn_3_O_4_–AL had not obviously changed, but the relative intensity of those peaks weakened, illustrating that part of the SiO_2_ and carbonate minerals had reacted. The diffraction peaks at 2θ = 45.4° and 57.6° were related to hausmannite (Mn_3_O_4_) (PDF No. 24-0734), which confirmed that Mn_3_O_4_ had been loaded onto the argillaceous limestone [15]. These results were consistent with those of XPS and FT-IR.

The other physiochemical characterizations of Mn_3_O_4_–AL are presented in Table S1. With modification, the pH and point of zero charge (PZC) were changed from 8.32 (AL) to 7.92 (Mn_3_O_4_–AL) and from 1.95 (AL) to 3.20 (Mn_3_O_4_–AL), respectively. The specific surface area was increased from 3.49 (AL) to 24.5 m^2^/g (Mn_3_O_4_–AL) which was consistent with the morphology of the Mn_3_O_4_–AL as evidenced by SEM (Figure 1a). Those changes were mainly ascribed to the addition of a large amount of acidic MnCl_2_ solution in the preparation process of Mn_3_O_4_–AL, which etched the surface of AL by the neutralization of some carbonate and hydroxyl functional groups. The increased specific surface area can expose more adsorption sites on the surface, which is conducive to the adsorption of heavy metals. In addition, the initial content of total Pb, Cu, and Ni in the Mn_3_O_4_–AL were 38.1, 12.1, and 20.9 mg/kg, respectively, which were below the environmental quality standard for soils in China (GB 15618-2018) [38]. Therefore, the environmental risk during application of Mn_3_O_4_–AL can be negligible. Moreover, the cation exchange concentration (CEC) of the Mn_3_O_4_–AL and AL were 31.5 and 5.87 cmoL(+)/kg, respectively. The higher CEC of the Mn_3_O_4_–AL could be ascribed to the increased exposure of ion exchange sites, which were not available before Mn modification. The increased CEC value of the Mn_3_O_4_–AL can further enhance the adsorption capacity of Pb^2+^, Cu^2+^, and Ni^2+^ [21].

### 3.2. Adsorption Behavior

#### 3.2.1. Adsorption Kinetics

**Figure 3:**
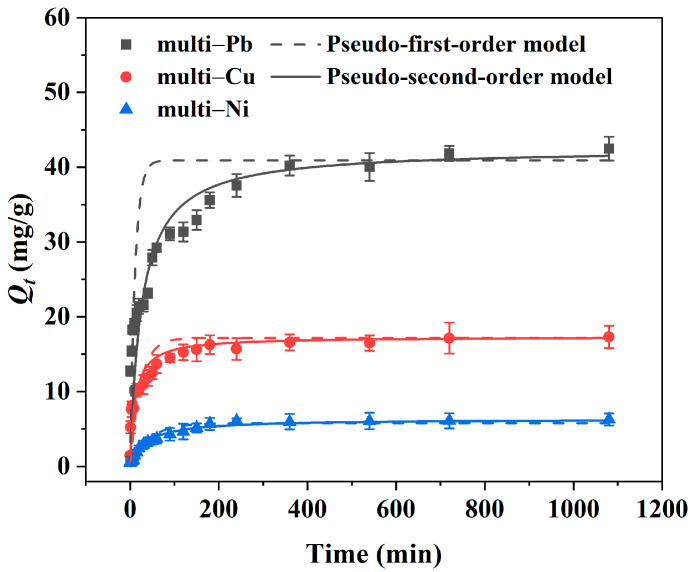
Adsorption kinetics and fitting curves of Pb^2+^, Cu^2+^, and Ni^2+^ onto Mn_3_O_4_–AL in multi-metal adsorption systems.

Adsorption kinetics is a significant indicator for evaluating the potential of adsorbents for practical applications. A rapid adsorption process could reduce the cost of remediation. The kinetics and fitting curves of Pb^2+^, Cu^2+^, and Ni^2+^ were investigated in a multi-metal system. The results are depicted in Figure 3, while the corresponding parameter values and correlation coefficients are presented in Table 1. Consistent with previous research, the adsorption rate of Pb^2+^, Cu^2+^, and Ni^2+^ in a multi-metal system increased rapidly during the first 120 min, followed by a slower adsorption stage before reaching equilibrium [39]. Finally, the adsorption equilibrium was reached after 360 min and the adsorption capacity stabilized at 42.48, 17.31, and 6.272 mg/g for Pb^2+^, Cu^2+^, and Ni^2+^, respectively. This was mainly attributed to the large number of adsorption sites on the Mn_3_O_4_–AL adsorbent and the higher driving force of concentration gradients in the adsorption primary stage of adsorption. As the free adsorption sites were gradually occupied and the repulsive force on the solid–liquid interface increased, the adsorption rate decreased until equilibrium was reached [21,40]. Compared to pseudo-first-order kinetics, the pseudo-second-order kinetic model fit the adsorption kinetic behavior of Pb^2+^, Cu^2+^, and Ni^2+^ on the Mn_3_O_4_–AL in a multi-metal system more closely, as indicated by the higher regression coefficient (R^2^). The theoretical equilibrium adsorption values (*Q_e_*) were closer to experimental values (Table 1). The results indicate that chemical adsorption may be the rate-controlling mechanism for the adsorption of Pb^2+^, Cu^2+^, and Ni^2+^ onto Mn_3_O_4_–AL in multi-metal systems.

#### 3.2.2. Adsorption Isotherm

**Figure 4:**
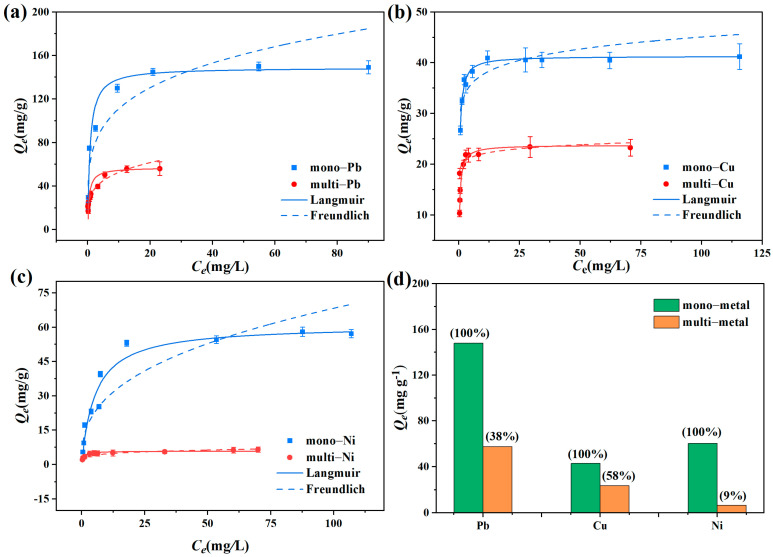
Adsorption isotherms of mono- and multi-heavy metal ions (**a**–**c**); Comparison of the maximum adsorption capacity of mono- and multi-heavy metal ions (**d**).

To gain information on the mechanisms of the adsorption effect, experiments on adsorption isotherms in mono- and multi-heavy metal systems were conducted. Figure 4 depicts the adsorption isotherms and fitting curves of the Langmuir and Freundlich models for adsorption of mono- and multi-heavy metal ions (Pb^2+^, Cu^2+^, and Ni^2+^) by the Mn_3_O_4_–AL. The corresponding parameters are shown in Table S2. In mono-metal systems, the R^2^ values obtained using the Langmuir model were higher than that of the Freundlich model, and the *Q_m_* for Pb^2+^, Cu^2+^, and Ni^2+^ were 148.73, 41.30, and 60.87 mg/g (Table S2), respectively. Compared to the Freundlich fitting results, the experimental data could be better fitted by the Langmuir model, suggesting the significant role of the monolayer adsorption process in the Mn_3_O_4_–AL adsorption of Pb^2+^, Cu^2+^, and Ni^2+^.

In the multi-metal system, the adsorption of Pb^2+^, Cu^2+^, and Ni^2+^ by the Mn_3_O_4_–AL could also be better described by the Langmuir model due to its higher R^2^ values compared to the Freundlich model. In addition, the *Q_m_* values of Pb^2+^, Cu^2+^, and Ni^2+^ were 56.96, 23.77, and 5.78 mg/g, respectively. These values represent 38%, 58%, and 9% of their values in mono-metal systems (Figure 4d). The adsorption selectivity of the Mn_3_O_4_–AL in the multi-metal system was in the order of Pb^2+^ > Cu^2+^ >Ni^2+^, indicating that the Mn_3_O_4_–AL exhibited better adsorption selectivity for Pb^2+^. Studies have shown that metal ions with higher electronegativity or a smaller hydrated radius exhibit a greater affinity for adsorption [41,42]. Metal ions with a smaller hydrated radius may have smaller steric hindrance during transport and adsorption to the inner surface of adsorbents [42]. The hydrated ionic radius and hydration energy of the three heavy metal ions were in the order of Pb^2+^ > Cu^2+^ >Ni^2+^ [41,43]. Evidently, the adsorption selectivity of the Mn_3_O_4_–AL was in accord with the hydrated ionic radius.

The maximum adsorption capacities of the Mn_3_O_4_–AL in this study were compared with those of other mineral adsorbents (Table S3) [21,23,29,44,45,46,47,48,49,50,51,52,53,54,55,56,57,58]. Although compared to CAL (chitosan-coated argillaceous limestone) or FAL (Fe^3+^-modified argillaceous limestone) [21,23], the Mn_3_O_4_–AL had lower adsorption of Cu^2+^ and Pb^2+^, the Mn_3_O_4_–AL had satisfactory adsorption of Ni^2+^. Furthermore, the adsorption capacities of the Mn_3_O_4_–AL for the three metals were greater than those of most natural and inorganically-modified mineral adsorbents, such as palygorskite, natural and Na-bentonite, goethite, Na-montmorillonite, and zeolite. Some organic material-modified minerals showed higher adsorption capacity than the Mn_3_O_4_–AL, such as cationic surfactant-modified bentonite, polyacrylamide/sodium montmorillonite, and sodium polyacrylate-grafted bentonite. However, the inorganic modifier has the advantage of being more cost-effective and naturally abundant compared to organic modifiers [21]. Therefore, the Mn_3_O_4_–AL is a promising adsorbent for removing heavy metals.

#### 3.2.3. Effects of Environmental Factors

**Figure 5:**
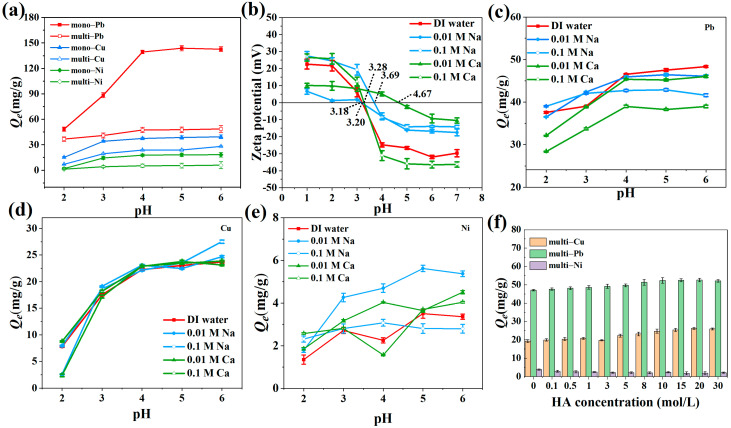
Effect of pH on the adsorption of mono- and multi-heavy metal ions (**a**); zeta potential of Mn_3_O_4_–AL at different ionic strengths and pH of solutions (**b**); Effect of ionic strength and pH on the adsorption of multi-heavy metal ions (**c**–**e**); Effect of HA on the adsorption process (**f**).

The initial solution pH is a significant factor influencing the adsorption process. Acidity can impact the surface charge of the adsorbent and adsorbate and ultimately affects the ability of the adsorbent to adsorb pollutants [42]. The pH of the solution may affect its surface charge due to the exchange of H^+^ [59]. Therefore, the effect of varying the initial solution pH on the adsorption capacity of the Mn_3_O_4_–AL was investigated to explore the adsorption mechanisms. Figure 5a shows the adsorption behaviors of mono- and multi-heavy metal ions (Pb^2+^, Cu^2+^, Ni^2+^) onto the Mn_3_O_4_AL from pH 2.0 to 6.0. The adsorption capacity (*Q_e_*) for Pb^2+^, Cu^2+^, and Ni^2+^ by the Mn_3_O_4_–AL increased from pH 2.0 to 4.0 and reached a plateau at a pH value of 6.0. At low pH, the adsorption of Pb^2+^, Cu^2+^, and Ni^2+^ was inhibited due to the competitive adsorption between H^+^ and Pb^2+^, Cu^2+^, and Ni^2+^ for adsorption sites [60]. In addition, Zeta potential analysis showed that Mn_3_O_4_–AL in deionized water carried a positive charge at pH levels below 4 (Figure 5b). Therefore, the electrostatic repulsion between the Mn_3_O_4_–AL and Pb^2+^, Cu^2+^, Ni^2+^ also resulted in a reduction of adsorption capacity. As the initial pH increased beyond the pH_pzc_, the surface charge of the Mn_3_O_4_–AL became negative [61]. Therefore, a stronger electrostatic attraction increases the adsorption capacity through electrostatic attraction [62]. In addition, the surface functional groups of the adsorbent were protonated at low pH, which weakened the complexation between the adsorbent and the heavy metal ions [61].

The adsorption of Pb^2+^, Cu^2+^, and Ni^2+^ by the Mn_3_O_4_–AL at different ionic strengths and alkali ions (i.e., Na^+^ and Ca^2+^) is presented in Figure 5c–e. The concentration of alkali ions had little effect on the adsorption of Cu^2+^ by the Mn_3_O_4_–AL. This is similar to the influence of ionic strength on Cu adsorption by Na-bentonite [63] and carbon nanotube-hydroxyapatite [64]. The adsorption capacity of Pb^2+^ and Ni^2+^ onto the Mn_3_O_4_–AL decreased with increasing concentrations of Na^+^ and Ca^2+^. A similar trend was reported in a previous study, where montmorillonite was employed for heavy metal adsorption [65]. This phenomenon mainly occurs because the adsorbed Pb^2+^ and Ni^2+^ may form outer-sphere surface complexes with the Mn_3_O_4_–AL. The electrolytes may compete with Pb^2+^ and Ni^2+^ for active sites on the Mn_3_O_4_–AL. In addition, the electrolytes may compress the electrical double layer thickness of the Mn_3_O_4_–AL, resulting in a decrease in the electrostatic attraction force between the Mn_3_O_4_–AL and heavy metal ions. This speculation could be supported by the higher inhibitory effect of Ca^2+^ as compared with Na^+^. Divalent ions can compress the thickness of the electric double layer more effectively than monovalent ions, preventing Pb^2+^ and Ni^2+^ from reaching the adsorbent surface [21]. Moreover, increasing the ionic strength may improve the aggregation of the Mn_3_O_4_–AL [66,67], which may further lead to a decrease in available adsorption sites of the Mn_3_O_4_–AL. In addition, the effects of different anionic concentrations were investigated and are shown in Figure S1. The concentration of Cl^−^ had little effect on the adsorption of Pb^2+^, Cu^2+^, and Ni^2+^ onto the Mn_3_O_4_–AL. The adsorption capacity of Pb^2+^, Cu^2+^, and Ni^2+^ on the Mn_3_O_4_–AL increased with increasing concentrations of SO_4_^2−^, primarily due to the formation of sulfate [23].

Humic acid (HA), a component of humic substances, is widely distributed in surface and groundwater. The ability of HA to complex with heavy metals can enhance the migration of heavy metal ions in solution and impact the removal of heavy metal ions [68]. Figure 5f illustrates the impact of HA on the heavy metal adsorption capacity of the Mn_3_O_4_–AL. The adsorption of Pb^2+^ and Cu^2+^ by the Mn_3_O_4_–AL increased as the concentration of HA increased, and reached a plateau at 15 mg/L. However, the adsorption of Ni^2+^ onto the Mn_3_O_4_–AL decreased as the concentration of HA increased. This distinct difference in the effect of HA can be explained by the following reasons. On the one hand, HA contains numerous functional groups, including phenols (–OH) and carboxylic groups (–COOH), which can offer extra adsorption sites and improve the adsorption of heavy metals [69]. Heavy metals can react with HA in a solution to create stable complexes [70,71]. Chaturvedi et al. (2007) also reported that humic acid can enhance the adsorption capacity of heavy metals on mineral surfaces through the formation of ternary mineral surface–metal–organic ligand complexes [72]. Therefore, the formation of Mn_3_O_4_–AL–HA–metal ion ternary complexes may enhance the adsorption of heavy metals. Research has shown that the affinities of these ions followed the order of Pb^2+^ > Cu^2+^ > Ni^2+^ [71]. The promotional effect of HA on the adsorption of Pb^2+^ and Cu^2+^ is greater than that of Ni^2+^. On the other hand, studies have shown that HA with a large size and low solubility, can accumulate at the solid/water interface to form a coating on the solid phase. HA attached to the solid phase surface will occupy adsorption sites and reduce the adsorption of heavy metals [73]. Therefore, we hypothesize that the HA adsorbed onto the Mn_3_O_4_–AL tends to combine with Pb^2+^ and Cu^2+^ rather than Ni^2+^ to form Mn_3_O_4_–AL–HA–Pb and Mn_3_O_4_–AL–HA–Cu complexes. This results in an increase in the adsorption capacity of Pb^2+^ and Cu^2+^ on the Mn_3_O_4_–AL with higher concentrations of HA, while the adsorption capacity of Ni^2+^ decreases.

**Figure 6:**
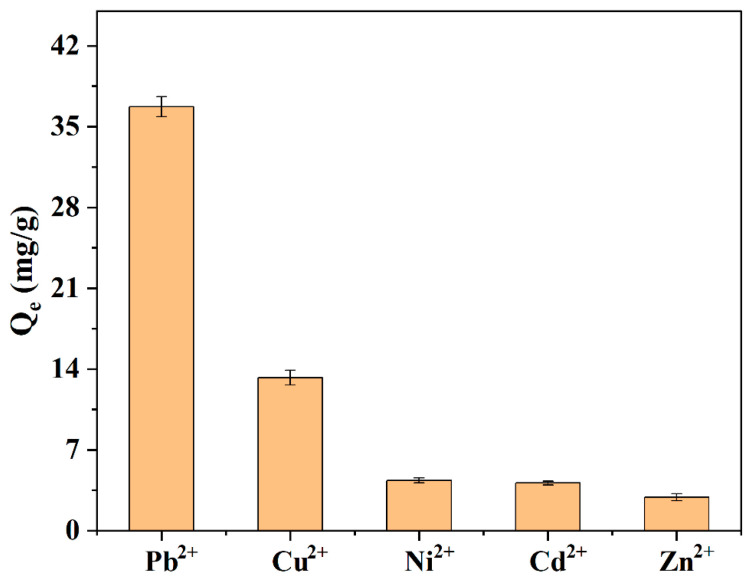
Comparison of the adsorption capacity of Pb^2+^, Cu^2+^, Ni^2+^, Cd^2+^, and Zn^2+^ in lake water-based simulated wastewater.

To simulate natural environmental conditions, lake water was used as the solvent to investigate the adsorption capacity of the Mn_3_O_4_–AL for Pb^2+^, Cu^2+^, Ni^2+^, Cd^2+^, and Zn^2+^ in a multi-metal system (Figure 6). Results showed that the adsorption capacity of Pb^2+^, Cu^2+^, and Ni^2+^ decreased to 36.72, 13.25, and 4.35 mg/g, respectively. The adsorption capacity of these heavy metals followed the order of Pb^2+^ > Cu^2+^ > Ni^2+^ > Cd^2+^ > Zn^2+^, which is consistent with previous research using montmorillonite and *Sphagnum* peat [41,65]. These results indicated that the Mn_3_O_4_–AL can effectively remove various heavy metal ions from lake water-based simulated wastewater.

#### 3.2.4. Adsorption Mechanisms

**Figure 7:**
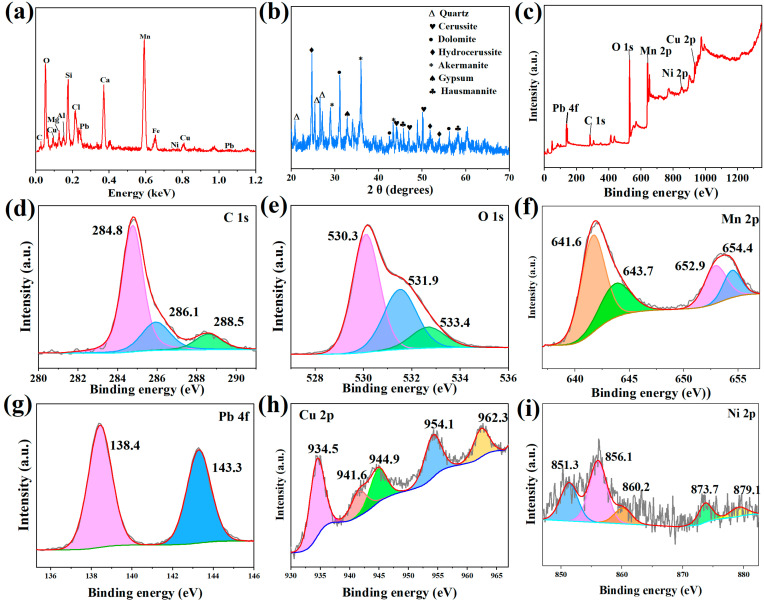
EDS images of Mn_3_O_4_–AL after competitive adsorption (**a**). XRD patterns of Mn_3_O_4_–AL after adsorption (**b**). XPS scan of Mn_3_O_4_–AL after competitive adsorption (**c**–**i**).

To gain further insight into the adsorption mechanisms of the Mn_3_O_4_–AL for Pb^2+^, Cu^2+^, and Ni^2+^, the chemical composition of the Mn_3_O_4_–AL after the adsorption process of multi-heavy metal ions (Pb^2+^, Cu^2+^, Ni^2+^) was determined using SEM-EDS, XRD, and XPS (Figure 7). Peaks of Pb, Cu, and Ni were obvious in the SEM-EDS curves after adsorption, indicating successful fixation of Pb, Cu, and Ni onto the Mn_3_O_4_–AL. The mineralogical composition of the Mn_3_O_4_–AL after competitive adsorption was characterized by powder XRD (Figure 7b). New diffraction peaks of hydrocerussite (2θ = 24.74°, 27.16°, and 51.0°) (PDF No. 28–0529) and cerussite (2θ = 44.1°, 48.94°, and 46.9°) (PDF No. 85–1088) appeared after adsorption, indicating the formation of Pb_3_(CO_3_)_2_(OH)_2_ and PbCO_3_ [21]. The results indicated that the precipitation of lead carbonate plays a crucial role in the process of the Mn_3_O_4_–AL adsorbing Pb^2+^.

The metallic state of elements in the Mn_3_O_4_–AL after competitive adsorptions was analyzed using XPS analysis (Figure 7c–i). The presence of Na, Mn, O, Ca, C, and Si elements in the Mn_3_O_4_–AL before competitive adsorption can be observed (Figure 2b). After competitive adsorptions, three new peaks Cu 2p, Ni 2p, and Pb 4f appeared in the Mn_3_O_4_–AL (Figure 7c), indicating the successful adsorption of Pb^2+^, Cu^2+^, and Ni^2+^ [19]. Meanwhile, the disappearance of Na 1s after competitive adsorption demonstrated that ion exchange was an important mechanism of the ability of the Mn_3_O_4_–AL to adsorb Pb^2+^, Cu^2+^, and Ni^2+^ [20,21]. The high CEC of the Mn_3_O_4_–AL (31.5 ± 1.82 cmoL(+)/kg) can be attributed in part to the presence of clay mineral components, which also confirmed that the Mn_3_O_4_–AL has a high ion exchange potential [21]. In addition, we proved that alkali ions can significantly influence the adsorption of Ni and Pb in the study mentioned above (Figure 5). This further confirmed that ion exchange was the primary adsorption process of competitive adsorptions. After adsorption, the C 1s peak was observed at 284.8, 286.3, and 288.8 eV (Figure 7d), which was consistent with the peaks observed before adsorption, indicating that the existing form of carbonate had negligible change after adsorption. Whereas, the O 1s peak changed from 529.9, 531.2, and 532.3 eV to 530.3, 531.9, and 533.4 eV. The O 1s peak shifted to a higher binding energy after adsorption. This shift in binding energy position may be due to electron transfer between the heavy metal ions (M^2+^) and the Mn_3_O_4_–AL, indicating the formation of –Mn–O–M^2+^, –Si–O–M^2+^ and –COO–M^2+^ compounds [21,74,75]. Furthermore, detailed spectra of the peaks of Pb, Cu, and Ni are shown in Figure 7. The main peaks of Pb 4f_7/2_ and Pb 4f_5/2_ were observed at 138.4 eV and 143.3 eV, indicating the formation of lead complex compounds during adsorption [76]. The Pb 4f_7/2_ peak occurred between the lead hydroxide (137.3 eV) and lead carbonate (138.7 eV) binding energy centers (Figure 7g), suggesting the formation of two lead species as a result of the adsorption by the Mn_3_O_4_–AL [76,77]. These findings were consistent with the XRD analysis results, which confirmed the formation of hydrocerussite (Pb_2_(OH)_2_(CO_3_)_2_) and cerussite (PbCO_3_). These results demonstrate that the precipitation of lead carbonate played a crucial role in the adsorption process of Pb^2+^ by the Mn_3_O_4_–AL. In the XPS spectra of Cu (Figure 7h), peaks at 941.6 and 944.9 eV were attributed to shake-up satellite peaks of Cu 2p_3/2_, which were caused by the charge transfer between Cu^2+^ and the ligand [77]. The peak at 962.3 eV confirmed the existing form of Cu(OH)_2_, while peaks at 934.5 and 954.1 eV were attributed to the formation of copper complex compounds, Mn_3_O_4_–AL–O–Cu [77]. Therefore, complexation and precipitation were the main mechanisms of Cu^2+^ adsorption [78]. As for Ni, the peaks at 856.1 and 873.7 eV corresponded to the peaks of Ni 2p_3/2_ and Ni 2p_1/2_, which were attributed to Ni(OH)_2_ [67,79]. The peaks that appeared at 860.2 eV belonged to NiO(OH) and 879.1 eV peak was attributed to the corresponding satellite peak of Ni 2p [67,80], indicating that precipitation was an important mechanism of Ni adsorption on Mn_3_O_4_–AL.

Furthermore, the introduction of negatively charged Mn_3_O_4_ makes AL more electronegative, leading to an electrostatic attraction between the Mn_3_O_4_–AL and heavy metal ions [81]. In addition, the increased specific surface area after Mn_3_O_4_ modification led to an increase in the number of contact sites on the Mn_3_O_4_–AL, which is favorable for the adsorption of Pb^2+^, Cu^2+^, and Ni^2+^.

In summary, the adsorption mechanisms of the Mn_3_O_4_–AL for Pb^2+^, Cu^2+^, and Ni^2+^ are as follows: (1) ion exchange between alkali ions and heavy metal ions; (2) precipitation to form PbCO_3_, Pb_2_(OH)_2_(CO_3_)_2_, Cu(OH)_2_, Ni(OH)_2_, and NiO(OH) due to the large proportion of carbonate and other basic functional groups in the Mn_3_O_4_–AL; (3) electrostatic interaction between the Mn_3_O_4_–AL and heavy metal ions due to the high negative charge of Mn_3_O_4_; and 4) formation of heavy metal complexes Mn–O–M^2+^, –Si–O–M^2+^ and –COO–M^2+^ by hydroxyl groups on the clay mineral components of the Mn_3_O_4_–AL. The Mn_3_O_4_–AL exhibited excellent performance in removing Pb^2+^, Cu^2+^, and Ni^2+^, which could significantly contribute to the remediation of heavy metal-contaminated water and soil.

## 4. Conclusions

In this study, we provided a strategy for the resourcization of argillaceous limestone modified with Mn_3_O_4_. The CEC and specific surface area increased significantly after modification. Mn_3_O_4_-modified argillaceous limestone has a high adsorption capacity for Pb^2+^, Cu^2+^, and Ni^2+^ in both mono-metal and multi-metal systems. At pH 2–4, the adsorption capacity increased with increasing pH values. At pH 4–6, the adsorption capacity was maximized and remained stable, indicating that the Mn_3_O_4_–AL is suitable for removing heavy metals in weakly acidic soil or neutral wastewater. As the concentration of HA increased, the adsorption of Pb^2+^ and Cu^2+^ increased, but the adsorption of Ni^2+^ decreased. Higher ionic strength was not conducive to the removal of Pb^2+^, and Ni^2+^, but it had no significant effect on the removal of Cu^2+^. Ion exchange, electrostatic interaction, precipitation, and complexation were the primary adsorption mechanisms. Of course, due to the limitations of laboratory-scale experiments and the intricacy of natural soil, further research is needed to assess the potential of the Mn_3_O_4_–AL in remediating heavy metal-contaminated soil.

## Figures and Tables

**Table 1:** Kinetic parameters of pseudo-first-order and pseudo-second-order models for competitive adsorption.

Adsorbates	Experimental *Q_e_* (mg/g)	Pseudo-First-Order Model			Pseudo-Second-Order Model		
		*Q_e_*	*k*_1_	*R^2^*	*Q_e_*	*k*_2_	*R* ^2^
		(mg/g)	(min^−1^)		(mg/g)	(g/(mg min))	
Pb	42.48	40.91	0.090	0.743	42.48	0.00092	0.928
Cu	17.31	17.17	0.043	0.895	17.32	0.00519	0.989
Ni	6.272	5.800	0.023	0.926	6.338	0.00449	0.979

## Data Availability

The data that support the findings are presented in this paper. Other data are available from the corresponding author upon reasonable request.

## Supplementary Materials

The following supporting information can be downloaded at: https://www.mdpi.com/article/10.3390/toxics12010072/s1, Figure S1: Effect of anionic concentration on the adsorption of multi-heavy metal ions on Mn_3_O_4_–AL; Table S1: Physicochemical characteristics of Mn_3_O_4_–AL; Table S2: Langmuir and Freundlich fitting parameters of mono- and multi-heavy metal ions adsorption isotherm on Mn_3_O_4_–AL; Table S3: Comparison of maximum adsorption capacities by Mn_3_O_4_–AL with other mineral adsorbents (with or without modification) reported in previous studies.
