# Supplementary material for: Resourcization of Argillaceous Limestone with Mn3O4 Modification for Efficient Adsorption of Lead, Copper, and Nickel
Source: Toxics. 2024 Jan 15;12(1):72. doi: 10.3390/toxics12010072 (PMC10820775; doi:10.3390/toxics12010072)
Supplement: Supplementary file 1 [file toxics-12-00072-s001.zip › toxics-2764248-SI.pdf]

Supplementary Material:

Resourcization of argillaceous limestone with Mn_3O_4 modification for efficient adsorption of Cu^{2+}, Ni^{2+}, and Pb^{2+}

Deyun Li[a, b], Yongtao Li[a, b], Tian Hu[b], Hanhao Li[a, b], Jinjin Wang[b], Zhen Zhang[b], Shuran He[c], Yulong Zhang[b, *]

[a] School of Environmental Science and Engineering, Shaanxi University of Science & Technology, Xi'an 710021, PR China.

[b] College of Natural Resources and Environment, Joint Institute for Environmental Research & Education, South China Agricultural University, Guangzhou 510642, China.

[c] College of Resource and Environment, Yunnan Agricultural University, Kunming，Yunnan 650201, China.

*Corresponding authors:

Yulong Zhang (email: yulongzhang@scau.edu.cn).

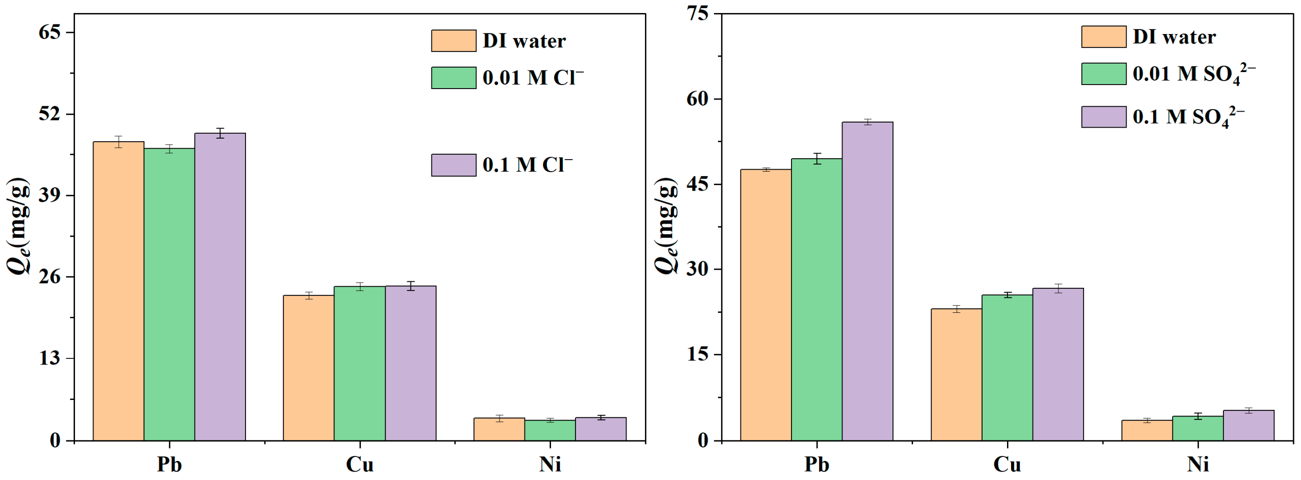

Figure S1 Effect of anionic concentration on the adsorption of multi-heavy metal ions on Mn_3O_4–AL.

Table S1 Physicochemical characteristics of Mn_3O_4–AL.

Contents	Mn_3O_4–AL
Total content of clay minerals (wt.%)	13.5 ± 0.16
pH	7.92 ± 0.48
Point of zero charge	3.20 ± 0.39
Cu (mg/kg)	12.1 ± 0.75
Pb (mg/kg)	38.1 ± 1.53
Ni (mg/kg)	20.9 ± 1.08
TOC (g/kg)	2.29 ± 0.71
DOC (mg/kg)	2.24 ± 0.19
BET surface area (m^2/g)	24.5 ± 3.03
Pore volume (cm^3/g)	0.12 ± 0.01
CEC (cmol (+)/kg)	31.5 ± 1.82

Table S2 Langmuir and Freundlich fitting parameters of mono- and multi-heavy metal ions adsorption isotherm on Mn_3O_4–AL.

Treatments	Langmuir			Freundlich		
	R^2	Q_m (mg/g)	K_L (L/mg)	R^2	$1/n$	K_F ($mg^{1-(1/n)}$ $L^{1/n}$/g)
Mono-Pb	0.95	148.73	0.76	0.85	0.23	65.09
Multi-Pb	0.95	56.96	0.37	0.94	0.23	31.35
Mono-Cu	0.98	41.30	0.35	0.71	0.08	31.52
Multi-Cu	0.96	23.77	0.39	0.65	0.05	19.77
Mono-Ni	0.98	60.87	5.26	0.87	0.37	12.42
Multi-Ni	0.98	5.78	0.69	0.93	0.19	0.19

Table S3 Comparison of maximum adsorption capacities by Mn_3O_4–AL with other mineral adsorbents (with or without modification) reported in previous studies.

Adsorbate	Absorbent	pH	Dosage (g/L)	Temperature (°C)	Initial concentration (mg/L)	Q_m (mg/g)	Reference
Cu	Mn_3O_4–AL	5.0	0.15	25	1–200	41.30	This study
	Citosan-coated argillaceous limestone	5.0	0.15	25	1–200	64.11	[22]
	Magnetic bentonite hydrogel beads	5.0	2	30	5–150	56.79	[43]
	Cationic surfactant modified bentonite	5.0	10	20	50–200	50.76	[29]
	Clinoptilolite	5.0	10	25	10–600	33.76	[44]
	Natural bentonite	5.0	1	30	5–250	32.26	[45]
	Surfactant modified montmorillonite	5.0	5	25	20–140	14.87	[46]
	Citosan-coated montmorillonite beads	4.0	3.33	25	10–200	13.04	[47]
	Iron-coated Australian zeolite	6.5	1–25	25	5–50	9.33	[48]
	Na-montmorillonite	5.5	25	20	0.21–4.14	8.45	[49]
	Palygorskite	5.0	10	25	0–100	2.356	[50]
Ni	Mn_3O_4–AL	5.0	0.15	25	1–200	60.87	This study
	Polyacrylamide/sodium montmorillonite	6.0	2	20	20–180	92.59	[51]
	Fe^{3+}-modified argillaceous limestone	5.0	0.15	25	1–200	50.9	[24]
	Chitosan-clay composite	4.5	4	20	50–800	32.36	[52]
	Natural bentonite	5.0	1	30	5–250	26.32	[45]
	Bentonites from Slovakia	5.9	5	40	50–300	21.93	[53]
	Acid-activated nanobentonites	–	12	25	50–350	14.41	[54]
	Na-bentonite	5.0	6.0	25	0–50	13.96	[55]
	Citosan-coated montmorillonite beads	4.0	3.33	25	10–200	12.18	[47]
	Palygorskite	5.0	10	25	0–100	0.481	[50]

Adsorbate	Absorbent	pH	Dosage (g/L)	Temperature (°C)	Initial concentration (mg/L)	Q_m (mg/g)	Reference
Pb	Mn_3O_4–AL	5.0	0.15	25	1–200	148.73	This study
	Citosan-coated argillaceous limestone	5.0	0.15	25	1–200	217.4	[22]
	Fe^{3+}-modified argillaceous limestone	5.0	0.15	25	1–200	184.4	[24]
	Clinoptilolite	5.0	10	25	50–2500	181.8	[44]
	Sodium polyacrylate-grafted bentonite	5.0	1.25	25	1100	149.62	[56]
	Mn-Substituted goethite	5.0	1	25	10–500	90.09	[57]
	Natural bentonite	5.0	1	30	5–150	85.47	[45]
	Na-montmorillonite	5.5	25	20	0.21–4.14	35.58	[49]
	Citosan-coated montmorillonite beads	4.0	3.33	25	10–200	29.85	[47]
	Iron-coated Australian zeolite	6.5	1–25	25	5–50	11.16	[48]
